# Safe Handling of Antineoplastic Drugs During Allergy Diagnostic Workup and Desensitization: A Single Experience of the Allergy Department in a Tertiary Hospital

**DOI:** 10.3389/falgy.2021.787537

**Published:** 2022-02-18

**Authors:** María Pilar Berges-Gimeno, Cristina Pueyo López, Alicia Barra-Castro, Emilio Solano Solares, Belén de la Hoz Caballer

**Affiliations:** ^1^Allergy Department, Ramón y Cajal University Hospital, Madrid, Spain; ^2^Instituto Ramón y Cajal de Investigación Sanitaria—IRYCIS, Ramón y Cajal University Hospital, Madrid, Spain; ^3^Red de Asma, Reacciones Adversas a Fármacos y Alergia, ARADyAL, Madrid, Spain; ^4^Pharmacy Department, Ramón y Cajal University Hospital, Madrid, Spain; ^5^School of Medicine, Alcalá University, Madrid, Spain

**Keywords:** desensitization, antineoplastic agent, safety handling, challenge drug, hazardous drugs

## Abstract

The increased use of antineoplastic drugs has been associated with a rising number of hypersensitivity reactions to these drugs, which has led to a growth in the demand for assistance from allergy services. The involvement of an allergist is essential to ensure that these patients with hypersensitivity reactions continue to be able to receive appropriate first-line treatment. Chemotherapy and biological agents have specific handling requirements and all the allergy departments involved in the diagnosis and therapeutic management of patients reacting to these drugs should find the means to guarantee safety. There are currently several guidelines on the safe handling of hazardous drugs for healthcare workers. However, specific recommendations are lacking for reducing occupational exposure in staff working in the allergy departments and managing these drugs for the diagnosis and management of hypersensitivity reactions. This review article focuses on the safe handling strategy of the allergy department in the Ramón y Cajal University Hospital and provides details of its implementation and experience over 10 years. This protocol could improve the knowledge of safe handling of antineoplastic drugs in allergy procedures.

## Introduction

Over the last two decades, referrals to the allergy departments for patients with confirmed/suspected drug hypersensitivity reactions (DHRs) to antineoplastic drugs have increased (1, 2). DHRs to antineoplastic drugs may lead to first-line drug avoidance, which can impact patient survival and quality of life (3). As the specialist trained to diagnose and risk stratifies DHRs, the role of the allergist is crucial to ensure personalized treatment. Rapid drug desensitization (RDD) is the cornerstone of the personalized therapeutic approach, and it is a safe and effective therapeutic tool to enable patients with allergies to receive their first-line treatment in spite of their hypersensitivity (4, 5).

The National Institute for Occupational Safety and Health (NIOSH) generally considers antineoplastic drugs as hazardous drugs (HDs) to workers in healthcare settings. HDs include any drug identified as hazardous or potentially hazardous on basis of at least one of the following criteria: carcinogenicity, developmental toxicity (including teratogenicity), reproductive toxicity, genotoxicity, and organ toxicity at low doses (6, 7). Research suggests that healthcare providers who handle HDs and who are exposed to even small amounts of certain drugs can be harmed, especially if exposure occurs continuously over years (8–10).

The number and variety of healthcare providers potentially exposed to HDs are on the rise because of the rapidly expanding use of these agents. Healthcare providers in the allergy departments have a potential risk of occupational exposure during the management of DHRs to antineoplastic drugs and during diagnostic allergy procedures [such as skin tests (STs), drug provocation tests (DPTs), and desensitization].

Over the past three decades, the NIOSH (6), the American Society of Health-System Pharmacists (ASHPs) (11), and the Oncology Nursing Society (ONS) (12) have published safety standards for healthcare professionals who handle potentially HDs in healthcare settings. However, to date, few recommendations for minimizing occupational exposure in the allergy department have been reported (4).

In 2010, the allergy department at the Ramón y Cajal University Hospital (RCUH) developed a program for the safe handling of HDs and for the management of antineoplastic drug-induced DHR from beginning to end. The program was based on existing national and international guidance documents (6, 8, 11–14) and adapted to the allergy department's work practices and organization.

This article provides a description of the program for the safe handling of HDs in the allergy department at the RCUH that includes risk assessment and safe strategies to minimize the exposure risk to HDs, with a focus on practical aspects of allergy diagnostic procedures and therapeutic measures.

## Implementing a Culture of Safe Handling of HDs in the Allergy Department

An increase in the number of patients referred to the RCUH allergy department for evaluation of DHRs to antineoplastic drugs led to the redesign of the department's clinical area for diagnostic and therapeutic procedures. The space was adapted to allow highly complex procedures to be carried out with maximum safety for patients and healthcare providers. In this technical area for diagnosis and therapeutic procedures, a dedicated care room for performing STs, DPTs, and desensitization to hazardous antineoplastic drugs (HADs) was included.

In addition, a program for the safe handling of HDs was developed and implemented with the aim of protecting the health of healthcare providers who could be exposed to HDs in their work practices. This program was the result of collaboration between members of a multidisciplinary team comprising an allergist, a pharmacist, a nurse, and occupational health physicians and was guided by national and international documents (6, 8, 11–14) and by institutional recommendations from the pharmacy department at the RCUH. Over the past 10 years, the model has been updated and improved in light of new recommendations and guidelines as well as accumulated experience with the large number of patients referred to the allergy department.

The program for the safe handling of HDs in the allergy department at the RCUH initially included a workplace-specific risk assessment that identified health and safety hazards; the best strategies to minimize occupational exposure were then sought (15).

## Hazard Assessment in the Allergy Department

The risk assessment must consider the inherent toxicity of a given drug and the degree of healthcare provider exposure (15). The basis of the assessment in the RCUH allergy department consisted of observation of work activities and provision of equipment and resources in the work areas where HDs were handled. The risk assessment process considered the following:

1. **Type of HDs handled**: Prior to handling any HD in the allergy department, knowledge of toxicity, including cytotoxicity, carcinogenicity, genotoxicity, teratogenicity, and organ toxicity at low doses, is required (6, 11, 15).
2. **Route of exposure**: Exposure can occur through dermal, inhalation, oral (e.g., contaminated foods, mouth contact with contaminated hands), and sharps/injections routes. Dermal absorption is generally regarded as the primary route of exposure in healthcare providers handling HDs (11) and is a route that becomes relevant during the reading of STs performed in the allergy department. Inhalation of aerosols and drug particles in the air, after pricking with the lancet during ST, also represents a problematic route of exposure to HDs in allergy healthcare providers.
3. **Risk of manipulation on work practices**: Healthcare providers and hospital staff in the allergy department who could potentially be exposed to HD include nurses, allergists, nursing assistants, orderlies, and workers involved in cleaning, transport, and waste disposal. Other healthcare providers involved in high-risk procedures (e.g., DPT and desensitization) performed in the intensive care unit (ICU) include intensive care physicians and nurses, as well as immunologists and research laboratory workers who develop *in-vitro* techniques with HADs.

The assessment of risk should take into account the degree of exposure of each healthcare professional or other staff according to the type of work activities carried out (15).

Nurses have the highest potential exposure to HDs, as they perform STs and administration of DPTs or RDDs. The nursing assistant can be quite exposed when handling body fluids or body fluid-contaminated clothing and linens. Allergists who come into direct contact with contaminated skin when they read the ST and need to palpate the papule may also be exposed to HDs.

## How to Prevent Occupational Exposure to HADs in the Allergy Department?

Healthcare providers in the allergy departments may be exposed to small doses of a broad range of cytotoxic everyday over the course of many years. Workplace-specific risk assessment and identification of HADs can help to define how best to protect health professionals and to establish strategies to reduce their exposure to HDs (15).

Since the initiation of the program for the safe handling of HDs in the RCUH allergy department, six strategies based on existing national and international guidance documents (6, 11, 12, 15) have been defined and implemented:

1. Creation of a specific list of HADs
2. Proper use of personal protective equipment (PPE)
3. Safe work practices during allergy procedures
4. Spill management
5. Hazardous drugs waste disposal
6. Competent personnel

### Creation of a Specific List of HADs

In the allergy department, frequently handled HDs include cytotoxic agents, biologic therapies, and disease-modifying targeted therapies for the treatment of cancer and non-malignant disease.

To identify those drugs requiring safe handling, we created a specific list of HADs based on the NIOSH list (6, 7) and endorsed by the pharmacy department. This list is dynamic, if a new agent is evaluated in the allergy department, the drug is reviewed and added to the list, if it meets the HD criteria. The list is reviewed at least every 12 months (15, 16, 22, 23).

The Allergy Department's current 2021 list of HADs includes drugs that frequently induce DHRs (Table 1): platins (oxaliplatin, carboplatin, and cisplatin) and taxanes (paclitaxel, docetaxel, and cabazitaxel). It also includes other chemotherapies that are less frequently related to reaction such as bendamustine, bleomycin, capecitabine, cyclophosphamide, doxorubicin, etoposide, fluorouracil, gemcitabine, irinotecan, methotrexate, and pemetrexed. This list also includes new antineoplastic agents associated with DHR: proteasome inhibitors (bortezomib), kinase inhibitors (crizotinib, dabrafenib, imatinib, regorafenib, sorafenib, and sunitinib), biologic response modulators (lenalidomide and pomalidomide), and recombinant fusion proteins that inhibit vascular endothelial growth factor (aflibercept) (6, 7).

**Table 1:** 2021 list of hazardous antineoplastic drugs (allergy department at the Ramón y Cajal University Hospital).

**Drug**	**Type antineoplastic drug**	**2016 NIOSH list classification (6)**	**2020 NIOSH list classification (7)**
Bendamustine	Alkilating drug	Group 1*	Group 1***
Bleomycin	Antibiotic cytotoxic	Group 1*	Group 1***
Bortezomib	Proteosoma inhibitors	Group 1*	Group 1***
Cabazitaxel	Antimicrotubule agent	Group 1 *	Group 1 ***
Capecitabine	Nucleoside metabolic inhibitor	Group 1*	Group 1***
Carboplatin	Alkilating drug	Group 1*	Group 1***
Crizotinib	Kinase inhibitors	Group 1*	Group 1***
Cisplatin	Alkilating drug	Group 1*	Group 1***
Cyclophosphamide	Alkilating drug	Group 1*	Group 1***
Dabrafenib	Kinase inhibitors	Group 1*	Group 1***
Docetaxel	antimicrotubule agent	Group 1*	Group 1***
Doxorrubicine	Anthracicline topoisomerase II inhibitor	Group 1*	Group 1***
Etoposide	Topoisomerase inhibitor	Group 1*	Group 1***
Fluoracil	Nucleoside metabolic inhibitor	Group 1*	Group 1***
Gemcitabine	Nucleoside metabolic inhibitor	Group 1*	Group 1***
Imatinib	Kinase inhibitors	Group 1*	Group 1***
Irinotecan	Camptothecin topoisomerase I inhibitor	Group 1*	Group 1***
Lenalidomide	Biologic response modulators	Group 2**	Group 1***
Methothrexate	Folate analog metabolic inhibitor	Group 1*	Group 1***
Oxaliplatin	Alkilating drug	Group 1*	Group 1***
Paclitaxel	antimicrotubule agent	Group 1*	Group 1 ***
Pemetrexed	Folate analog metabolic inhibitor	Group 1*	Group 1***
Pomalidomide	Biologic response modulators	Not included	Group 1***
Regorafenib	Kinase inhibitors	Group 1*	Group 1***
Sorafenib	Kinase inhibitors	Group 1*	Group 1***
Sunitinib	Kinase inhibitors	Group 1*	Group 1***
Aflibercept	Recombinant fusion protein that VEGF	Not included	Group 2**** Only met the NIOSH criteria as a developmental and/or reproductive hazard

*VEGF, vascular endothelial growth factor; NIOSH, National Institute for Occupational Safety and Health*.

Group 1* 2016 NIOSH list classification (6): antineoplastic drugs that meet one or more of the NIOSH criteria for hazardous drugs including those with manufacturer's safe handling guidance (MSHG).

Group 2** 2016 NIOSH list classification (6): antineoplastic drugs that meet one or more of the NIOSH criteria for hazardous drugs including those with MSHG.

Group 1*** 2016 NIOSH list classification (7): drugs that contain manufacturer's special handling information (MSHI) in the package insert and/or meet the NIOSH definition of hazardous drug and are classified by the National Toxicology Program (NTP) as “known to be carcinogenic” and/or classified by the International Agency for Research Cancer (IARC) as “carcinogenic” or “probably carcinogenic.”

Group 2**** 2020 NIOSH list classification (7): drugs that meet one of more NIOSH criteria for the hazardous drugs, but are not the drugs, which have MSHI or are classified by the NTP as “known to be carcinogen” or classified by the IARC as “carcinogenic” or “probably carcinogenic,” some of which also have adverse reproductive effects for the population at risk.

*NTP: National Toxicology Program (US Department of Health and Human Services): http://niels.nih.gov/pubhealth/roc/index.html*.

Monoclonal antibodies (mAbs) do not seem as hazardous as cytotoxic drugs, due to their mechanism of action and their large molecular weights, which would be expected to limit bioavailability and toxic potential. Recently, the NIOSH (7) declared that mAbs do not meet the criteria for HDs, although occupational exposure risks associated with these drugs have not been widely studied and are unclear. While evidence regarding the risk of chronic low-grade occupational exposure during the handling of mAbs is limited, a cautious approach to handling and disposal should be recommended (17).

### Proper Use of PPE

Personal protective equipment provides a barrier between the worker and HDs during episodes of potential contact. Appropriate PPE must be worn when handling HDs during admission, skin testing, administration of DPT and RDD, decontamination and cleaning, spill control, and waste disposal. PPE requirements should be protocolized for each activity in which HDs are handled (6, 11, 15).

The protective equipment used for handling HDs in the allergy department should be made available to all the healthcare providers and should include the following (6, 11, 15):

- **Gloves:** Gloves are essential when handling HDs. Gloves should comply with the American Society for Testing and Materials (ASTMs) standard D6978 (18) and must be powder free. Gloves should be inspected for visible defects before use and should be removed at the end of the procedure or if they are visibly damaged or contaminated. Proper hand hygiene must be practiced before and after removing gloves. Wearing double gloves provide an additional protective barrier (6, 11, 15). Two pairs of gloves are required for skin testing and for cleanup of HD spills.
- **Gowns:** Gowns should be disposable and made of the material tested to be protective against HDs. Chemotherapy gowns should have greater protection in the area of sleeves and front. HD-handling gowns should include long sleeves with tight-fitting cuffs and should close at the back. Gowns must be changed at the end of any procedure and in the event of contamination, spillage, or rips (6, 11, 15).
- **Safety goggles:** Appropriate eye protection should be worn when there is a risk of splashing, when performing STs, and when cleaning up a spill. Safety goggles should be anti-splash and watertight. Face shields alone do not provide full eye and face protection (6, 11, 15).
- **Respiratory protection masks:** Respiratory masks should provide a barrier to splashes, droplets, and sprays around the nose and mouth. Surgical masks do not provide adequate respiratory protection. Recommended respiratory protection devices are the N95 respirator mask (13, 15) or a more protective respirator mask (6, 11). The FFP3 mask is used at the RCUH.

### Safe Work Practices During Allergy Procedures

Avoidance of hazards requires safe work practices within specific workplaces. In addition to proper use of PPE and safe work environments, there must exist a stringent work practice program that includes training and demonstrated competence of each worker involved in each activity that involves the management of HADs (6, 11).

#### Designation of HD Areas for Allergy Diagnostic and Therapeutic Procedures

Allergy diagnostic and therapeutic procedures related to HADs should be performed in places that guarantee safe handling of HADs (e.g., spill kits, waste containers) (6, 15).

The RCUH's technical area for diagnostic and therapeutic procedures in the allergy department includes a specific care room (the “premium room”) where allergy workers perform skin testing, non-risk DPT, and desensitization to HADs. This premium room is fully equipped as an intermediate care unit and contains the necessary resources for the safe handling of HADs: two spill kits, the HDs waste container, and an exclusive bathroom for patients. The optimal environment for skin testing with HDs should be a negative pressure side room or an alternative as deemed appropriate by the occupational health and pharmacy departments. The latter is the alternative in our center with the premium room. Considering the specific preparation of drugs for skin testing in the hospital pharmacy, see below, and the use of PPE, our room, with the above recommendations, provides a safe environment, which can be adapted to each allergy department.

All these areas and processes were risk assessed in our hospital by pharmacy and occupational health departments.

High-risk DPT and desensitization are carried out in the ICU, where health professionals are well-trained in the safe handling of HDs and have a spill kit and an HD waste container readily available (2, 4). The allergy department has two beds in the ICU supervised by an intensivist and an allergist.

#### Safe Practices for the Allergy Procedures

Every allergy department must have written a safe work practices protocol that addresses all the aspects of safe handling of HDs during allergy diagnostic and therapeutic procedures (6, 19). This plan should include standard operating procedures (SOPs) and should be readily available and accessible to all professionals including temporary employees (19). These SOPs should address admission, skin testing, administration *via* DPT or RDD, and handling of HD waste.

##### Skin Testing for HAD

During skin prick testing (SPT) for HADs, there is a potential risk of inhalation or splashing due to droplets that are generated after pricking the drop of pure or diluted HAD with the lancet. In addition, when reading STs (SPTs and intradermal tests), there is a risk of dermal absorption *via* contact with the skin contaminated with a HAD.

Skin tests for HADs should be carried out in a designated room with a spill kit and an HD waste container. Preferably, only the patient and the nurse performing the ST should stay in this room.

**Figure 1:**
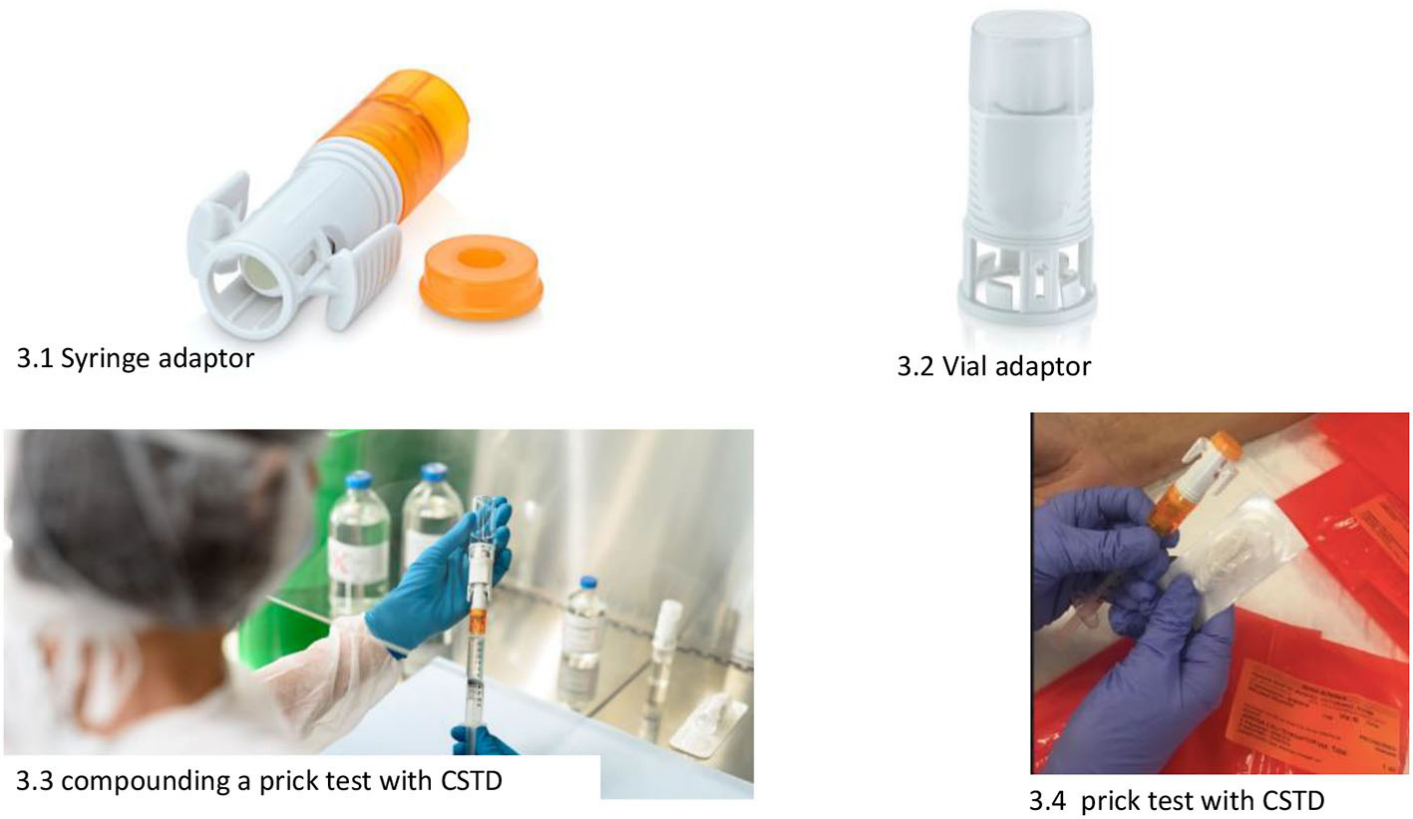
CSTDs: Tevadaptor® (photos used with the permission of the manufacturer).

Skin tests are prepared in syringes equipped with closed system transfer devices (CSTDs) (6) (Figure 1). This is to prevent (1) the generation of aerosols in the preparation; (2) accidental spills (since the syringe plunger remains airtight until an adapter is attached at the time of the test); and (3) the possible contamination of allergy service personnel when performing the tests.

**Procedures and PPE**

**Figure 2:**
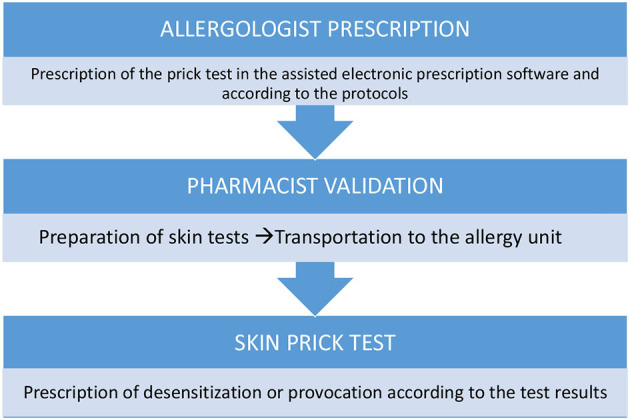
Skin test work-flow.

a) All the necessary items for skin testing should be placed in the work area before testing begins.
b) Skin tests for HAD must be prepared by the pharmacy department in class IIb, biological safety cabinets (BSCs) (Figure 2), and in syringes with CSTDs (Luer-lock fittings). When STs are performed, the needles are connected to the syringe through the Luer-lock fittings (Figure 1).
c) When receiving the HAD syringes, the healthcare provider must wear only one pair of chemotherapy gloves and must ensure that there is no external damage on the packaging.
d) The healthcare provider handling the HAD for an ST must be adequately trained.
e) The healthcare provider must wear proper PPE, including double chemotherapy gloves (ASTM D6978), a protective gown, a self-filtering mask (FFP3), and safety glasses.
f) A plastic-backed absorbent pad should be placed under the work area and above the work table during the ST and should be changed at the end of the ST.
g) After performing an ST, residues of the HAD droplets should be dragged through the drying pad, which should then be discarded immediately.
h) Hazardous drug syringes with the safety needle attached and lancets must be placed in sharps containers and then in a cytotoxic waste container along with other contaminated materials.
i) The healthcare providers should remove their PPE immediately after completing the procedure to avoid spreading contamination. Specific procedures for PPE removal must be established and followed (11).
j) Personal protective equipment used for skin testing should be placed in a sealable plastic bag for containment before disposal as contaminated waste.

##### Administration (DPT/Desensitization)

Antineoplastic treatments with cytotoxic drugs, such as chemotherapy desensitization or DPT, must be prepared, according to current national legislation, in the pharmacy departments of hospitals, in the area of chemotherapy preparation of the same. This area is a negative pressure clean room, with an anteroom where handling personnel can wash their hands and put on PPE [double chemotherapy gloves (ASTM D6978)], chemotherapy gown, self-filtering mask (FFP3), and safety goggles (6, 11, 15) before entering. In the clean room in the RCUH, there are three class IIb, BSCs (6, 11, 15). These provide vertical laminar airflow and are designed to protect personnel, the products prepared, and the environment.

Closed system transfer devices (6, 11, 15) are used in the preparation and subsequent conditioning of the treatments to be dispensed to the allergy department. A CSTD is a drug-transfer device that mechanically prohibits the transfer of environmental contaminants into the system and the escape of HD or vapor concentrations outside the system (Figure 1).

**Figure 3:**
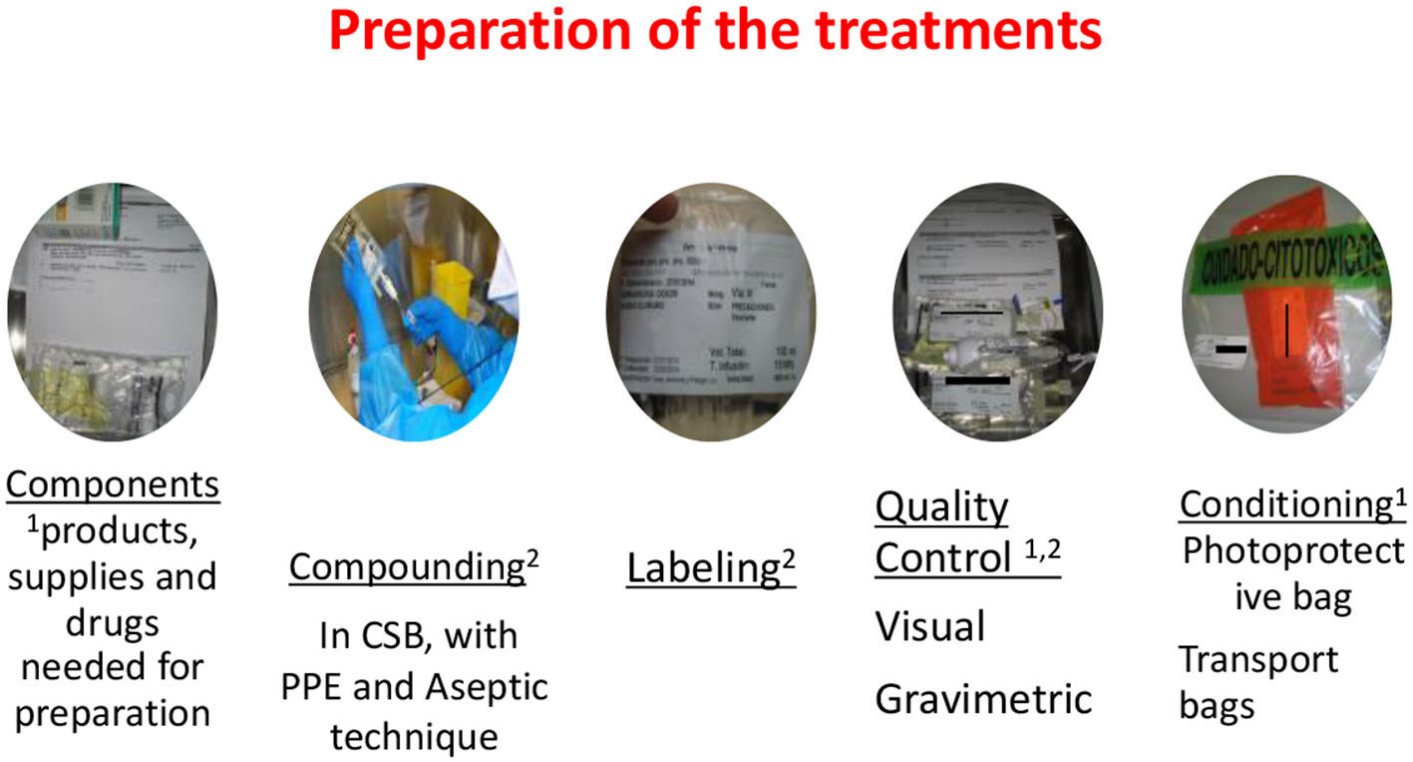
Preparation of the treatments: (1) Pharmacy technician (2) Pharmacy nurse.

Bags of the provocation and desensitization treatments are dispensed with the extension set already attached and purged with the diluent. At the administration site, the treatment only has to be connected through a Luer-lock connection to the infusion system of the patient. Pharmacy technicians bag the treatments, which are individualized for each patient. An orderly transports the bags to the allergy department in a dedicated chemotherapy transport car to increase safety and prevent accidental spills and contamination (Figure 3).

**Drug provocation test** is the controlled administration of a drug to study DHRs. In the RCUH, DPT is performed in the ICU beds assigned to the allergy desensitization program, where the desired full dose of the culprit drug is prepared in a single infusion bag and administered according to the instructions of the manufacturer (20). An expert group of allergists, intensive care physicians, and nurses, trained in the proper administration, containment, and disposal of HDs, oversees the procedure. The ICU area has a spill kit and HD waste container. Access to the administration area is limited to patients receiving therapy and essential personnel only.

**Figure 4:**
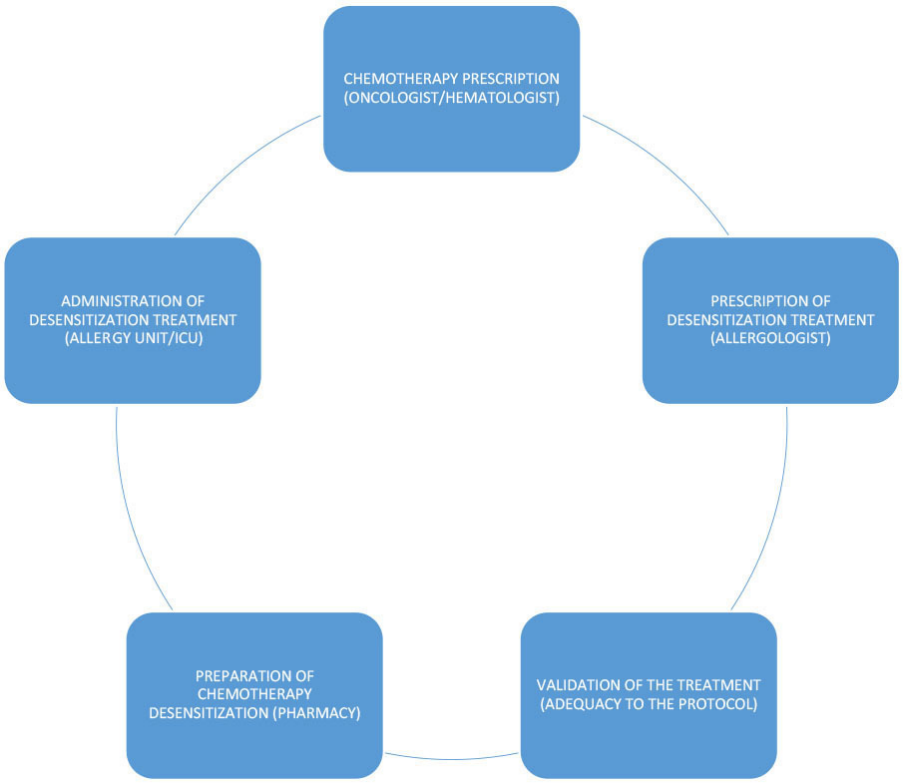
Desensitization treatments work-flow.

**Rapid drug desensitization** is a drug-specific procedure that induces a temporary hyporesponsive state by an incremental escalation of suboptimal doses (21). RDD protocols may include three or four infusion bags. All the desensitization treatments must be previously and collaboratively protocolized by the allergy and the pharmacy departments (Figure 4). Protocolization increases patient safety and reduces the risk of possible errors. Only protocolized preparations are made. The preparation of desensitization treatments takes longer than the preparation of standard therapy, due, among other reasons, to the need to prepare intermediate solutions of the active principle. It is essential to consider this necessary extra time when scheduling preparation in the safety cabinets.

High-risk desensitizations are performed in the ICU and non-risk desensitizations are carried out in the technical area for diagnostic and therapeutic procedures. Most DPTs and RDDs with HADs are done intravenously. Below, we detail how both the procedures are performed with the safe handling of HAD in mind.

**Administration (DPT/RDD) Procedures and PPE**

a) When receiving the infusion bags, the healthcare provider should wear chemotherapy gloves (ASTM D6978) and should examine visually the bags and verify that they are intact.
b) Appropriate PPE should be worn and should include gloves (ASTM D6978) and (intravenous administration without CSTDs) if splash protection is required, a protection gown, a self-filtering mask (FFP3), and safety goggles (11).
c) Locking connections should be used to securely connect the bag-attached extension set to the intravenous tubing. During desensitization, successive bags are connected to multiple infusion sets following the established protocol.
d) The intravenous system should be flushed with non-drug solutions before removal. It should then be discarded in its entirety with bags, without making disconnections (11, 15).
e) Access to a spill kit and HD waste container must be available (11, 15).
f) Equipment such as an administration infusion set with bags and PPE must be properly disposed off in HD waste containers after administration.

#### *In vitro* Tests

*In-vitro* tests, such as basophil activation test (BAT) or lymphocyte transformation test (LTT), should be performed in a class IIb, BSC with vertical laminar air flow. Immunologists and research laboratory workers should wear adequate PPE: double chemotherapy gloves (ASTM D6978), chemotherapy gown, self-filtering mask (FFP3), and safety goggles. HAD for *in vitro* tests must be prepared by the pharmacy department in syringes with CSTDs. A spill kit and HD waste container must be available (11, 15). BAT and LTT are techniques that are not validated.

### Spill Management

A written action plan to control and cleanup HD spills and spill kits must be readily available in all the areas where HDs are routinely handled. Spill kits should contain all of the materials needed to clean HD spills: two pairs of gloves (ASTM D6978), one HD-resistant gown, one pair of disposable HD-resistant booties, one cap, one pair of splash goggles, one protective FFP3 mask, one absorbent plastic-backed sheet, one disposable towel, at least two sealable thick plastic hazardous waste disposal bags, one dustpan, and one pair of tweezers (11).

If a spill occurs, access to the spill area should be immediately restricted and a spill kit should be immediately made available. Trained personnel wearing appropriate PPE must immediately clean any spill. All the contaminated items and PPE should be discarded in cytotoxic waste containers. Personnel who were potentially exposed during the spill require immediate evaluation (11, 15). The circumstances and management of HD spills should be documented.

### Hazardous Drug Waste Disposal

The term “HD waste” includes any material that makes contact with an HD during a procedure (e.g., drug bags, tubing, lancets for ST, syringes, and protective equipment). All the patient waste (urine, blood, and feces) should be considered to contain the drugs or their metabolites and should be handled as hazardous waste. An HD waste container should be available in the workplace (6, 11, 15). An HD waste should be placed in a waste container that is clearly marked with a cytotoxic hazard symbol. Anyone who handles HD-contaminated waste and body fluids should wear double chemotherapy gloves and a protective gown. They should also wear face/eye protection when there is a risk of splashing and respiratory protection if there is potential for inhalation (6).

### Competent Personnel

Professionals involved in the administration of HDs must have the appropriate skills. Before an HD is introduced into a new clinical setting, appropriate knowledge acquisition and training within the field of practice should be implemented and the competency of relevant personnel must be reassessed at least every 12 months (15, 16, 22, 23).

In the US, chemotherapeutic agents may only be administered by *chemotherapy certified nurses*. However, in Spain, there is no such certification, so all the care providers involved in the procedure received specific training on the handling of cytotoxic.

The program for the safe handling of HDs in the RCUH allergy department includes theoretical and practical training for healthcare providers prior to them handling HDs. The pharmacist of our multidisciplinary team is the Board Certified Oncology Pharmacist (BCOP) by the Board of Pharmacy Specialties (BPSs). All the allergy healthcare providers are specifically evaluated by occupational health physicians before working with HAD and monitored every 12 months.

All the professionals involved in handling these drugs such as nurses and allergists as well as other support staff such as nursing assistants, cleaners, and porters are trained. All must demonstrate competency every 12 months. The training includes (6, 15):

– Knowledge of the Allergy Department's list of HADs
– Correct work practices (education and training relevant to each person's role in the department and type of work)
– Proper use of PPE
– Spill management
– Proper HD waste disposal.

## Conclusion

When the allergy department includes the diagnostic and therapeutic management of DHRs to antineoplastic drugs in its clinical practice, it should implement a culture of safe handling of HADs. Occupational risk to healthcare providers in the allergy department should be assessed and recommended safe practices for skin testing, administration (DPTs/RDDs), spill management, and HD waste disposal should be established.

The program for the safe handling of HDs in the allergy department at the RCUH is a program based on standards in published guidelines and on the experience of over 10 years of HAD management. This model of safe handling could serve as a useful tool to guide and improve safety practices in the other allergy departments. More research is needed to determine the level of risk associated with the handling of HADs in the allergy departments and to define optimal safety strategies.

## Safe Practices for the Handling of HDs: Key Points

- The implementation of a culture of safe handling of HDs is essential in the allergy department when it begins to address the management of DHRs to these agents.
- Occupational risks to healthcare workers in the allergy department depend on the toxicity of the antineoplastic drugs, the types of exposure, and how these drugs are handled in work practices.
- Inhalation and dermal absorption are routes of exposure to consider when performing SPTs.
- The allergy department should develop and maintain the HADs list in the allergy department, which helps to recognize the drugs with potential toxicity.
- The designation of specific areas for handling HDs, the proper use of PPE, and the implementation of safe work practices to avoid health hazards are essential strategies for the protection of healthcare providers.
- Hazardous antineoplastic drug allergy workup should be performed in locations that guarantee safe handling (e.g., ready access to spill kits and HD waste containers).
- Healthcare providers and staff who handle HDs must be trained according to their job functions.

## Data Availability Statement

The original contributions presented in the study are included in the article/supplementary material, further inquiries can be directed to the corresponding author/s.

## Ethics Statement

The studies involving human participants were reviewed and approved by Comité de Ética de la Investigación del Hospital Universitario Ramón y Cajal. The patients/participants provided their written informed consent to participate in this study. The RCUH Ethics Committee approved the study (institutional register: 273/12).

## Author Contributions

MB-G and CP designed the manuscript, reviewed all the procedures, which are described, and wrote the draft of the article, that was later reviewed by the rest of the authors of the paper. MB-G, AB-C, and ES carried out the study for the diagnosis, with the performance of drug provocations and desensitization treatments of the patients. BH and MB-G have coordinated and supervised the development of the entire manuscript. All authors contributed to the article and approved the submitted version.

## Funding

MB-G reports grants from Instituto Carlos III (Spanish Government) (Scholarship file number: PI 18/00962) and from Fundación Merck Salud 2019 during the conduct of the study.

## Conflict of Interest

The authors declare that the research was conducted in the absence of any commercial or financial relationships that could be construed as a potential conflict of interest.

## Publisher's Note

All claims expressed in this article are solely those of the authors and do not necessarily represent those of their affiliated organizations, or those of the publisher, the editors and the reviewers. Any product that may be evaluated in this article, or claim that may be made by its manufacturer, is not guaranteed or endorsed by the publisher.
